# Laser-Scribed Graphene on PDMS for Flexible Wearable Sweat Biosensors with Multiplexed Sensing Capability

**DOI:** 10.3390/bios16050277

**Published:** 2026-05-11

**Authors:** Aida Rakhimbekova, Lavita Nuraviana Rizalputri, Aris Konstantinidis, Saptami Suresh Shetty, Khaled Nabil Salama

**Affiliations:** 1 Bioengineering Program, Biological and Environmental Science and Engineering Division, King Abdullah University of Science and Technology (KAUST), Thuwal 23955-6900, Saudi Arabia; aida.rakhimbekova@kaust.edu.sa (A.R.); lavita.rizalputri@kaust.edu.sa (L.N.R.); aris.konstantinidis@kaust.edu.sa (A.K.); saptami.shetty@kaust.edu.sa (S.S.S.); 2 Electrical and Computer Engineering Program, Computer, Electrical and Mathematical Science and Engineering Division, King Abdullah University of Science and Technology (KAUST), Thuwal 23955-6900, Saudi Arabia

**Keywords:** wearable biosensor, sweat analysis, multiplexed sensing, laser-scribed graphene (LSG), polydimethylsiloxane (PDMS), ion-selective electrodes

## Abstract

Sweat is a valuable biofluid for non-invasive health monitoring, as it contains electrolytes, metabolites, and organic compounds that can correlate with blood levels, making it highly attractive for wearable sensing. Building on advances in low-cost, portable electrochemical sensors, sweat analysis enables tracking of hydration status, metabolic stress, and energy availability via key markers such as sodium, potassium, lactate, and glucose. In the sports context, such wearable platforms can support performance optimization and recovery by assessing fluid loss and electrolyte balance in real time. Here, a multiplexed wearable sweat patch is developed to simultaneously monitor temperature, pH, ammonium, sodium, and sweat rate. The integrated platform demonstrates sensitivities of 10.1 mV/ln[NH_4_^+^], 9.1 mV/ln[K^+^], 1.11 mV/ln[Na^+^], 14 mV/pH, 0.19% °C^−1^, and approximately −1.0 mA (mL/min)^−1^ for sweat rate, with stable signals and linear calibration responses over relevant physiological ranges. The sensor is implemented on a lightweight, biocompatible laser-scribed graphene on a PDMS substrate suitable for prolonged skin contact and mechanical deformation. In addition, a custom PDMS adhesive patch with optimized suction-cup microstructures is engineered to improve skin adhesion under both dry and wet conditions. Finally, the design of the platform was inspired by an adaptive cycling marathon across Saudi Arabia, where an earlier prototype of a wearable patch was deployed for real-time monitoring during a 30-day campaign.

## 1. Introduction

Among various biofluids, sweat is especially abundant in analytes that closely reflect blood concentrations, making it a highly suitable medium for wearable sensor applications [1]. Sweat is predominantly composed of water, but it also contains key analytes such as electrolytes, metabolites, and organic compounds, making it a rich source of biochemical information [1,2]. Detection methods include fluorescence sensing, colorimetric methods, and electrochemical sensing, with electrochemical sensing being popular due to its lower cost, good performance, and transportability [2]. Elevated levels of certain components, such as chloride and sodium, can serve as diagnostic markers for conditions like hypovolemia, while other substances, such as glucose, lactate, and ammonia, can provide insights into metabolic conditions [3]. The use of wearable sensors to monitor sweat composition offers a promising avenue for continuous, real-time health monitoring, particularly in the context of sports and fitness [4,5]. Sweat analysis is crucial, providing a non-invasive method to assess biomarkers that guide training and recovery strategies [6,7,8,9,10]. Many studies now concentrate on hydration, deploying sensors that track fluid loss and electrolyte levels as key gauges of an athlete’s physiological status [5,6,11]. Hydration is particularly essential for optimal performance [12], whereas lactate levels indicate metabolic stress and help adjust training [13,14,15,16]. Furthermore, glucose levels in sweat are shown to reflect energy availability for endurance [16,17,18].

In recent years, significant advancements have been made in the development of multiplexed wearable sensors for comprehensive health monitoring [7,19]. For instance, in 2016, Wei Gao and his team introduced a wearable sensor array capable of simultaneously detecting multiple electrolytes in sweat. Their device demonstrated sensitivities of 64.2 mV per decade for sodium (Na^+^) and 61.3 mV per decade for potassium (K^+^), both exhibiting near-Nernstian behavior. Additionally, the integrated temperature sensor showed a linear response with a sensitivity of approximately 0.18% per degree Celsius within the physiological skin temperature range of 20–40 °C [19].

Furthermore, various graphene-based sensors have been explored for measuring temperature in wearable sensors. In the context of thermal sensing, Tiina Vuorinen et al. (2016) demonstrated graphene/PEDOT:PSS sensors with sensitivity of −0.068% °C^−1^ [20], while Liu et al. (2022) achieved enhanced sensitivity of 1.60% °C^−1^, linearity (R^2^ = 0.99), and an accuracy of 0.3 °C by doping graphene with polyaniline (PANI) on a PDMS substrate [21].

Salvo et al. reported that the sensitivity of graphene-based pH sensors can range from 12 to 99 mV/pH [22]. A notable example is the work of Žutautas et al. (2023), who utilized laser-induced graphene (LIG) functionalized with polyfolate (PFA), resulting in a pH sensitivity of 30.32 ± 0.50 mV/pH [23]. Our multiplexed sensor aims to integrate these capabilities into a single platform, providing comprehensive monitoring of physiological parameters relevant to health and performance.

The integration of multiple analyte-specific detection mechanisms into a single, flexible, and wearable platform is essential for enabling real-time, comprehensive sweat analysis with high temporal resolution. Among the various material combinations investigated for this purpose, the coupling of laser-scribed graphene (LSG) with polydimethylsiloxane (PDMS) presents notable advantages. PDMS is widely recognized for its mechanical flexibility, biocompatibility, and suitability for skin-interfacing applications, while LSG offers a cost-effective and scalable fabrication route with excellent electrical and electrochemical properties [24]. To facilitate the incorporation of LSG onto flexible substrates such as PDMS, several transfer methodologies have been developed. Zhu et al. (2024) reported the use of a spin-coated mixture of PDMS, polyimide (PI) particles, and polyethyleneimine (PEIE), followed by CO_2_ laser scribing, resulting in an LSG film with a conductivity of 80 μA [25]. Mahmood et al. (2020) demonstrated the fabrication of LSG on kraft lignin using a CO_2_ laser, followed by direct transfer onto PDMS, achieving a capacitance of 880.25 μF cm^−2^ (equivalent to approximately 176.9 nA) [26]. Antonelli et al. (2024) employed a method involving LSG formation on PI, solvent-assisted transfer onto PMMA, and the application of heat and pressure, yielding a conductivity of 15 μA [27]. These approaches illustrate the ongoing development of practical and efficient techniques for integrating conductive graphene-based elements into flexible bioelectronic systems.

In this work, we present a proof-of-concept wearable sweat patch based on laser-scribed graphene transferred onto a flexible PDMS substrate, integrating multiple electrochemical sensing modules within a single platform. The device combines temperature, pH, ammonium, sodium, and sweat-rate sensing with a custom-engineered PDMS adhesive patch featuring suction-cup microstructures to enhance skin adhesion under both dry and wet conditions. The electrochemical performance of the transferred LSG electrodes is characterized by cyclic voltammetry, ion-selective calibration, and pH potentiometry, while the mechanical robustness of the patch is evaluated through systematic adhesion tests on skin-like substrates. Finally, we demonstrate the feasibility of the system in a real-world scenario by deploying the patch during an adaptive cycling marathon across Saudi Arabia, highlighting its potential for continuous sports monitoring and rehabilitation-oriented applications.

## 2. Materials and Methods

### 2.1. Materials and Apparatus

Polydimethylsiloxane (PDMS) component A and component B, polyvinyl chloride (PVC), tetrahydrofuran (THF), Potassium ionophore I—cocktail B (Selectophore™), Ammonium ionophore I—cocktail A (Selectophore™), Sodium ionophore X (Sigma Aldrich, St. Louis, MO, USA), Dioctyl Sebacate (di(2-ethylhexyl)sebacate) 95%.

Commercial polyimide (PI) sheets were purchased from Utech Products (Schenectady, NY, USA). Potassium Ferrocyanide (K_4_[Fe(CN)_6_]), Potassium Ferricyanide (K_3_[Fe(CN)_6_]), and Potassium Chloride (KCl) were purchased from Fisher Chemical (Geel, Belgium). Phosphate-buffered saline (PBS) tablets were purchased from MP Biomedicals (Santa Ana, CA, USA). All aqueous solution studies employed ultrapure water from a Milli-Q^®^ integrated water purification system (Merck KGaA, Darmstadt, Germany; resistivity: 18.2 MΩ.cm at 25 °C). pH sensor (Fisher Scientific accumet^®^ AB150).

### 2.2. LSG Electrode Fabrication

The fabrication process for the sweat sensor involved the direct application of conductive, porous graphene electrodes onto commercial Polyimide (PI) films, as reported in previous research [28], each with a thickness of 125 microns. This application was carried out using an infrared CO_2_ laser with a wavelength of 10.6 microns and a laser spot diameter of approximately 150 microns (Universal Laser Systems^®^ PLS6.75) under ambient conditions. To achieve optimal adhesion of the carbon mesh to the substrate and attain a porous morphology, the laser parameters were set to 2.7% power, a speed of 4.5 cm/s, 1000 pulses per inch, and a laser-to-surface distance of 2 mm.

### 2.3. PDMS Patch Fabrications

The preparation of two Polydimethylsiloxane (PDMS) mixtures. Initially, a pre-polymer base and a curing agent (SYLGARD^®^) are mixed in a 20:1 and 5:1 ratio by weight, respectively. These mixtures are thoroughly stirred for several minutes to achieve homogeneity [29]. Following this, the mixtures are subjected to degassing under vacuum for 45 min to remove any entrapped air bubbles.

### 2.4. LSG on PDMS Transference

A 5:1 PDMS ratio is applied to an LSG polyimide sheet to cover the pattern, then the mixture is vacuumed for 15 min. After that, the LSG polyimide sheet is preheated in an oven at 90 °C for 10 min and then fixed onto a 3D-printed mold (Figure S1). The PDMS mixtures with a 20:1 ratio are poured onto a 3D-printed mold. The mold is then placed in an oven and cured at 90 °C for 45 min. After curing, the solidified PDMS is carefully peeled off from the mold and PI sheet, keeping LSG on it [30].

### 2.5. Temperature Sensor Fabrication

The temperature sensor is based on a pure LSG patterned on a PDMS substrate. The LSG serves as the active sensing material, where its electrical resistance changes in response to temperature variations due to graphene’s conductivity following a two-dimensional continuous medium model, where carrier mobility rises with temperature, leading to a negative exponential resistance-temperature relationship [31]. The sensor device features a wavy design to reduce strain effects during bending or stretching, ensuring stable and reliable temperature measurements, particularly for wearable applications [32].

### 2.6. pH Sensor Fabrication

This study explores a pH sensor utilizing graphene functionalized with gold (Au) and zinc oxide (ZnO), leveraging graphene’s exceptional conductivity and large surface area as a substrate. ZnO’s semiconducting properties and biocompatibility make it ideal for selectively interacting with hydrogen ions (H^+^) in various pH environments.

The gold solution was prepared by dissolving 170 mg of HAuCl4 in 10 mL of 0.5 M HCl. Then, the gold was electrodeposited on an LSG electrode using chronoamperometry with a potential of −0.9V for 240 s. The 0.1 M Zinc nitrite solution was prepared and heated up on a hot plate to 70 degrees. Using three electrode systems: Working Electrode (WE)—LSG electrode, Reference Electrode (RE)—Ag/AgCl, Counter Electrode (CE)—Pt; Zinc was electrodeposited using Chronoamperometry with a potential of −1 V for 900 s.

### 2.7. Ion-Selective Sensor Fabrication

The ion-selective membranes for both potassium and ammonium sensors were prepared using a similar procedure. First, 3.28 mg of polyvinyl chloride (PVC) was dissolved in 100 µL of tetrahydrofuran (THF) under continuous stirring for 30 min to ensure complete dissolution. To enhance ion selectivity, 7.65 µL of Potassium ionophore I—cocktail B (Selectophore™) was added for the potassium sensor, while 7.65 µL of Ammonium ionophore I—cocktail A (Selectophore™) was used for the ammonium sensor [33].

Since the ion-selective membranes for sodium sensors were prepared using a solution of 33 mg of polyvinyl chloride (PVC) in 1000 µL of tetrahydrofuran (THF), which was stirred for 30 min. Then, 66 mg of dioctyl sebacate (DOS) plasticizer was added to the PVC-THF solution and stirred until completely dissolved. Finally, 2 mg of Sodium ionophore X was added to the sodium sensor [19,33]. The resulting membrane solutions were then applied to the WE of tLSG on PDMS via drop-casting, with 1 µL per drop, for a total of four drops. Each drop was allowed to dry at room temperature (21 °C) for 2 h before the next application to ensure proper layer formation and membrane stability.

The calibration was conducted using a PalmSens4 potentiostat (PalmSens BV, Houten, The Netherlands), in open circuit potential (OCP) mode. Each concentration was added sequentially, and the potential was recorded for 200 s per step.

### 2.8. Adhesive Patch from PDMS

All molds were printed (Phrozen Sonic Mighty 8K) using thermostable resin (TR300) to prevent shrinkage and size shift during PDMS curing. Afterward, molds were additionally cured overnight in the oven at 120 °C.

The patch was made from PDMS using a different pre-polymer base and a 20:1 curing agent ratio [24]. PDMS was poured into a 3D printed mold, vacuumed for 15 min, then covered with an acrylic cup with LSG on a PI sheet fixed on it and cured in an oven at 90 °C for 45 min.

### 2.9. Sweat Rate Sensor Fabrication

A sweat rate sensor was fabricated by integrating interdigital electrodes (IDE) made from laser-scribed graphene (LSG) with a microfluidic PDMS layer. The LSG-IDE structure was patterned on a polyimide substrate using CO_2_ laser scribing, forming comb-shaped electrodes with interspacing widths of 500 µm. A PDMS microchannel (0.2 mm width × 0.4 mm height × 4.5 mm length; ~0.36 µL volume) was fabricated using a 3D-printed mold and bonded atop the IDE layer.

## 3. Results

### 3.1. Design and Characterization of Transferred LSG

**Figure 1:**
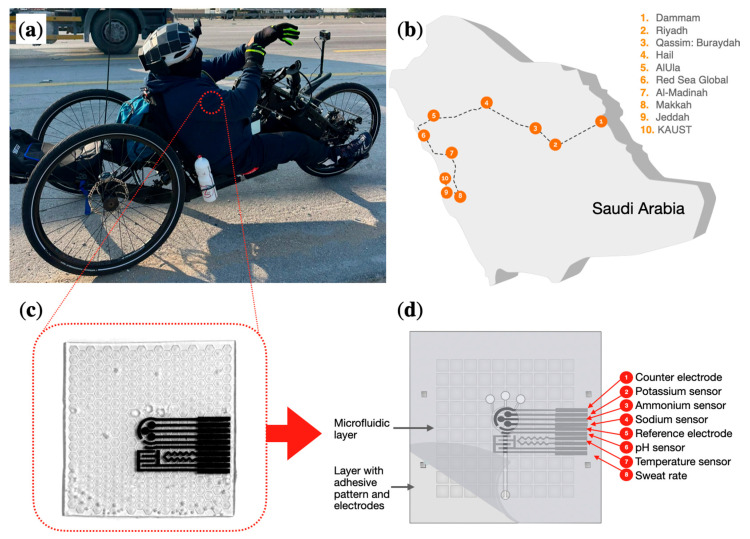
Overview of the multiplexed wearable sweat patch and field deployment: (**a**) Adaptive hand-cycling scenario and location of patch placement. (**b**) Route of the 30-day cycling marathon across Saudi Arabia [34]. (**c**) Photograph of the assembled device. (**d**) Schematic of the patch architecture showing the tLSG electrodes, PDMS adhesive layer, microfluidic channels, and electronics module.

**Figure 2:**
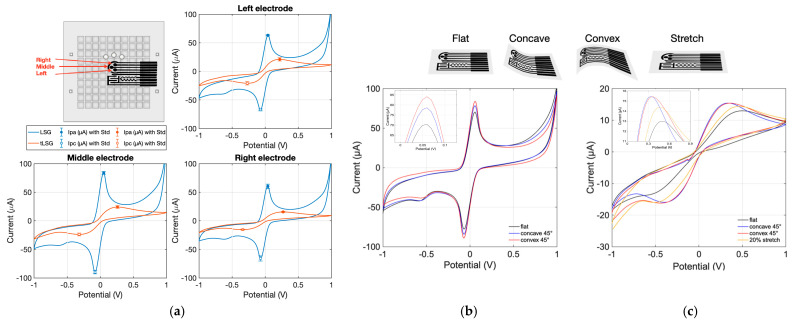
Electrochemical performance and mechanical robustness of tLSG electrodes: (**a**) Cyclic voltammograms of LSG and tLSG electrodes in 10 mM [Fe(CN)_6_]^3−^/^4−^ with 10 mM PBS. (**b**) CVs of LSG on PI in flat, concave (45°), and convex (45°) configurations. (**c**) CVs of tLSG on PDMS under flat, concave (45°), convex (45°), and 20% transverse stretching, demonstrating preserved voltammetric response under deformation.

A cost-effective and reproducible technique was developed for transferring laser-scribed graphene (LSG) from polyimide (PI) onto PDMS substrates to form the functional electrode layer of the wearable patch. The overall device layout is illustrated in Figure 1, which shows the adaptive hand-bike use case, the route of the cycling campaign, the assembled device, and the general patch architecture with sweat inlets and alignment features. The electrical properties of the transferred LSG (tLSG) electrodes were characterized by cyclic voltammetry (CV) in a ferro/ferricyanide redox probe using a PalmSens potentiostat. Each measurement was carried out at a scan rate of 0.05 V/s over a potential window from −1 V to +1 V in 1 mV steps in 10 mM [Fe(CN)_6_]^3−^/^4−^ with 10 mM PBS as the supporting electrolyte (Figure 2a).

The resulting CV curves were used to extract anodic (Ipa) and cathodic (Ipc) peak currents for the left, middle, and right electrodes (Table 1). Variations in peak currents were attributed primarily to geometric differences and the relative positioning of the electrodes with respect to the reference and counter electrodes in the cell configuration. While cathodic peak currents generally exhibited higher absolute values, anodic peak currents were more consistent across devices and were therefore selected as the main metric for subsequent comparison. Overall, the transfer method yielded tLSG electrodes with an average electrical conductivity of approximately 0.06 S/m and a relative variation of about 5%, indicating good reproducibility and stable electrochemical behavior suitable for integration into the multiplexed sweat patch (Figures S2–S4).

The mechanical robustness of the electrodes under bending and stretching was further evaluated by cyclic voltammetry. For LSG on PI, the CV curves recorded in the flat, concave (45°), and convex (45°) configurations almost overlapped, with only a slight increase in peak current (<10%), indicating that moderate bending does not significantly affect the electrochemical behavior of the as-scribed electrodes (Figure 2b).

For tLSG on PDMS, bending (concave and convex, 45°) and transverse stretching up to 20% produced only moderate changes in the CV response, characterized by a 20–25% increase in peak current amplitude and a slight shift in the redox peaks towards more positive potentials, while the overall CV shape and reversibility were preserved (Figure 2c). These variations suggest minor changes in effective resistance and interfacial contact under deformation, while the overall voltammetric shape and reversibility are preserved. Together, these results confirm that the transferred LSG electrodes maintain their electrochemical functionality under bending and stretching deformations representative of on-skin wearable use.

### 3.2. Morphological Characterization

**Figure 3:**
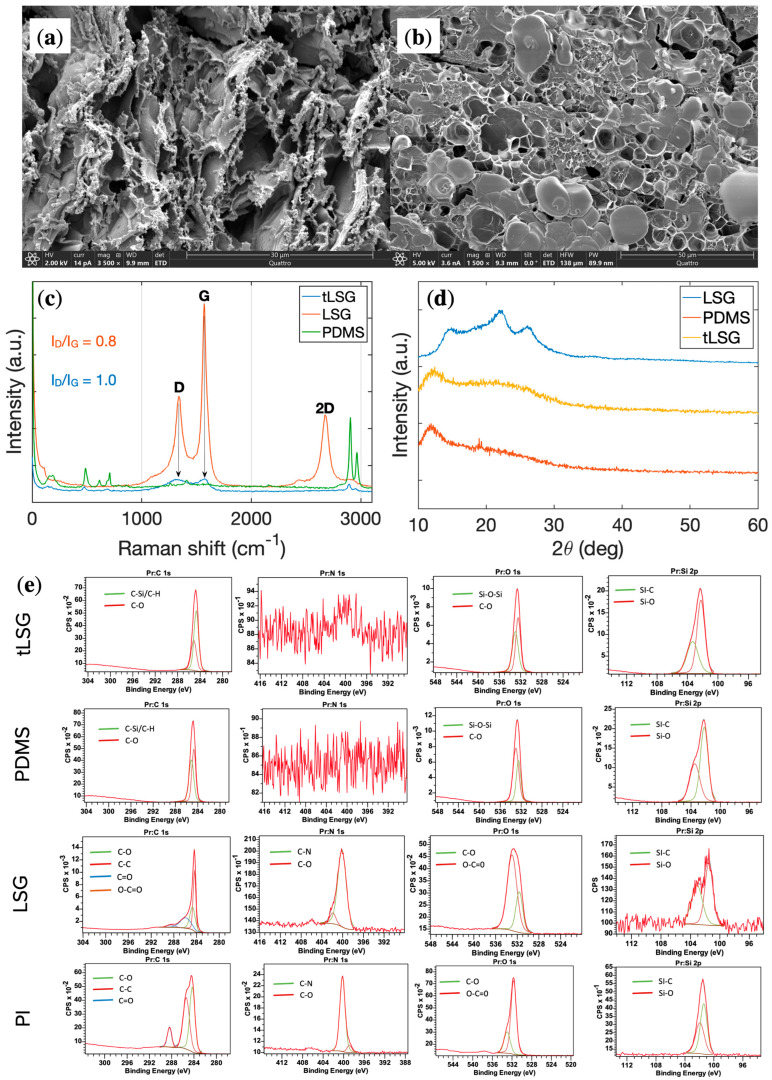
Morphological and structural characterization of LSG before and after transfer: (**a**) SEM image of as-scribed LSG on PI showing a porous, interconnected graphene network. (**b**) SEM image of tLSG on PDMS with a smoother exposed surface due to layer inversion. (**c**) Raman spectra of LSG and tLSG; the increased $I_{D}/I_{G}$ ratio after transfer indicates a higher defect density. (**d**) XRD patterns of PI, LSG on PI, PDMS, and tLSG on PDMS; the disappearance of a distinct (002) graphene peak and the presence of a broad 20–30° feature are consistent with a disordered, few-layer graphene structure embedded in the polymer matrix. (**e**) High-resolution XPS spectra of C 1s, N 1s, O 1s and Si 2p for tLSG, PDMS, LSG and PI surfaces, showing deconvoluted components associated with graphitic carbon, oxygenated carbon, nitrogen functionalities and silicon–oxygen/carbon bonds after laser scribing and transfer.

Once LSG was patterned and transferred onto PDMS, both the as-scribed LSG electrodes (LSGE) and the transferred LSGE (tLSGE) structures were examined using a Quattro SEM system equipped with energy-dispersive X-ray spectroscopy (EDS). Prior to imaging, a thin 2 nm iridium coating was applied to improve conductivity and image quality. As shown in Figure 3a, the as-scribed LSGE displays a highly porous (≈3–5 μm), interconnected network typical of laser-scribed graphene, which increases the effective surface area and facilitates charge transfer in electrochemical applications. After transfer onto PDMS (Figure 3b), the exposed surface appears smoother and less porous because the transfer process inverts the graphene layer, such that the originally unscribed backside faces outward and lacks the same ablated texture.

EDS mapping confirms this structural rearrangement by detecting silicon signals from the underlying PDMS together with carbon, nitrogen, and oxygen associated with the graphene network (Figure S5). Raman spectra acquired with a 532 nm excitation (Figure 3c) show characteristic D, G, and 2D bands for LSG at approximately 1340, 1570, and 2670 cm^−1^, respectively, in agreement with previous reports on laser-scribed graphene. After transfer to PDMS, these bands remain present, but the D/G intensity ratio increases from 0.8 to 1.0, indicating a moderate rise in defect density likely induced by the transfer and interaction with the elastomer surface. The PDMS spectrum exhibits its typical vibrational features, confirming preservation of the polymer matrix [35,36].

Complementary XRD analysis further supports this picture (Figure 3d). The as-fabricated LSG on PI displays a discernible graphitic feature associated with the substrate and the (002) graphene reflection near 22–26°, whereas in the transferred LSG on PDMS, this peak becomes strongly weakened or indistinguishable. Instead, tLSG shows only a broad scattering hump in the 20–30° region that is more pronounced than for bare PDMS, consistent with a disordered or poorly crystalline graphene phase with reduced coherent stacking and thickness embedded in the polymer matrix [37,38]. High-resolution XPS provides additional insight into the surface chemistry of these films (Figure 3e). For LSG directly written on PI, deconvolution of the C 1s region reveals an enhanced sp^2^ C–C/C=C contribution accompanied by a relative decrease in oxygenated carbon species, in line with partial graphitization of the PI surface. In contrast, the spectra of tLSG on PDMS closely resemble those of bare PDMS: both are dominated by Si–C/Si–O components in the Si 2p and O 1s regions, and the C 1s envelope is characteristic of siloxane rather than graphene-like carbon, while no detectable N 1s signal is observed, indicating the absence of residual PI at the probed surface. These XPS results imply that the transferred LSG layer is extremely thin compared to the XPS sampling depth and/or partially buried under a reorganized PDMS overlayer, so that the polymer signal overwhelms the graphene contribution, yet the PDMS chemistry remains unchanged, corroborating that the transfer does not chemically degrade the elastomer. Taken together, the loss of a sharp (002) reflection in XRD, the increased $I_{D}/I_{G}$ ratio in Raman, and the substrate-dominated XPS response on PDMS indicate that the transfer step yields a few-layer, defect-rich graphene network that is embedded within and partially masked by the PDMS matrix rather than being removed, which is still sufficient to sustain the stable electrochemical performance described in Section 3.1.

### 3.3. Temperature Sensor

The sensing mechanism of the graphene-based temperature sensor relies on monitoring changes in electrical resistance as temperature varies [39]. As temperature increases, enhanced phonon scattering perturbs charge-carrier transport in graphene, leading to a reproducible increase in resistance [39,40]. This thermosensitive behavior enables detection of small temperature fluctuations with high sensitivity, which is attractive for wearable and biomedical applications [16,41].

**Figure 4:**
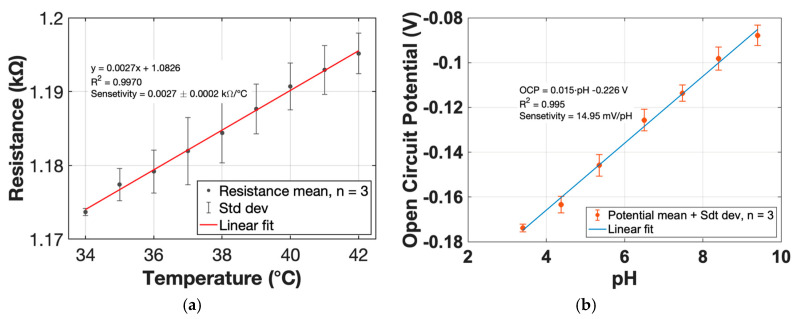
Temperature and pH sensing characteristics: (**a**) Resistance–temperature response of the LSG temperature sensor between 34–42 °C, with a sensitivity of 2.7 Ω/°C (0.19% °C^−1^) and temperature coefficient of resistance (TCR)—2 × 10^−3^; (**b**) Potentiometric calibration of the ZnO/Au/graphene pH sensor, showing a sensitivity of 14.95 mV/pH and $R^{2}=0.99$.

The fabricated LSG temperature sensors demonstrated a high degree of reproducibility across three replicates, with an average sensitivity of 2.7 ± 0.24 Ω/°C (n = 3) in the physiologically relevant range of 34–42 °C (Figure 4a). The positive and approximately linear resistance–temperature relationship confirms stable and predictable thermoresistive behavior suitable for on-skin operation. The calculated temperature coefficient of resistance (TCR = 0.00229 °C^−1^) falls within the range reported for flexible carbon-based thermistors, indicating good thermal responsivity of the LSG material [42]. The low variance between devices further suggests robust fabrication and supports scalable production of temperature channels for integrated sweat patches.

### 3.4. pH Sensor

The working principle of the pH sensor is based on the interaction between hydrogen ions (H^+^) and the ZnO layer electrodeposited on a graphene electrode functionalized with gold. ZnO, a semiconducting metal oxide, responds to changes in H^+^ concentration by modulating its surface charge, which alters the interfacial potential at the electrode–electrolyte boundary [43]. This shift is recorded as a change in the open-circuit potential (OCP), enabling potentiometric determination of pH. The underlying graphene/Au layer provides high conductivity and a large effective surface area, which together improve signal stability and charge-transfer characteristics [43,44].

Experimental calibration revealed a robust linear response within the tested pH range, with a sensitivity of 14 mV/pH determined from the slope of the OCP versus pH regression (OCP = 0.014·pH + 0.347 V) and a coefficient of determination, $R^{2}=0.99$, confirming high linearity (Figure 4b). The observed sensitivity corresponds to approximately 24% of the theoretical Nernstian slope (59.16 mV/pH at 25 °C), which is consistent with many ZnO-modified electrodes operating in near-neutral media. Despite the sub-Nernstian response, the sensor exhibits stable and reproducible potentiometric behavior, making it suitable for tracking relative pH changes in sweat during prolonged wearable operation.

### 3.5. Ion-Selective Sensors

To detect ammonium, potassium, and sodium ions in sweat, ion-selective membranes (ISMs) were employed on top of the tLSG electrodes. However, due to the known cross-selectivity of ammonium-selective membranes toward potassium ions, which are also present in sweat at comparable levels, both species were monitored in parallel to ensure more accurate interpretation of the ammonium signal.

For sensor calibration, solutions covering the physiological concentration ranges of ammonium, potassium, and sodium in sweat were prepared in phosphate-buffered saline (PBS). The ammonium calibration was performed using six NH_4_^+^ concentrations (0.1, 1, 5, 10, 50, and 100 mM) together with a PBS control. Potassium and sodium calibration solutions were prepared with six concentrations each (1, 5, 10, 25, 50 mM for K^+^ and 1, 10, 25, 50, 75, 100 mM for Na^+^), again including PBS as a blank, reflecting typical sweat ranges of 2–30 mM for potassium and 10–90 mM for sodium.

**Figure 5:**
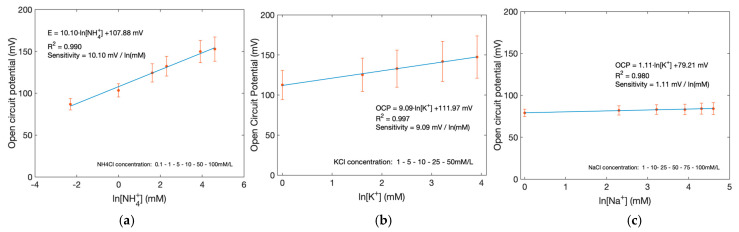
Ion-selective potentiometric responses: (**a**) Calibration of the NH_4_^+^-selective electrode with a sensitivity of 10.1 mV/ln[NH_4_^+^]. (**b**) Calibration of the K^+^-selective electrode with a sensitivity of 9.1 mV/ln[K^+^]. (**c**) Calibration of the Na^+^-selective electrode, which exhibits a strongly sub-Nernstian slope of 1.11 mV/ln[Na^+^], indicating the need for membrane optimization. Full OCP versus ln[ion] datasets are provided in Figures S6–S8.

The ammonium-selective electrode exhibited a sensitivity of 10.1 mV/ln[NH_4_^+^], while the potassium-selective electrode showed a sensitivity of 9.1 mV/ln[K^+^], both with good linearity over the tested ranges. In contrast, the sodium-selective electrode reached only 1.11 mV/ln[Na^+^], which is far below the theoretical Nernstian response of approximately 58.9 mV/ln[Na^+^] at 21 °C. The open-circuit potential (OCP) versus ln[ion] calibration curves for NH_4_^+^, K^+^, and Na^+^ are provided in Figure 5, respectively, and illustrate the distinct slopes and dynamic ranges obtained for each ion-selective channel.

The reduced performance of the sodium-selective electrode can be attributed to several factors, including suboptimal membrane composition or thickness, insufficient loading of the sodium ionophore and/or anionic additive, incomplete THF evaporation during casting, or poor membrane adhesion on the hydrophobic tLSG surface, which may have impaired ion-to-electron transduction. As a result, the Na^+^ channel in this proof-of-concept platform is considered preliminary and mainly serves to demonstrate feasibility, whereas the NH_4_^+^ and K^+^ channels show more reliable potentiometric behavior suitable for further optimization toward quantitative sweat analysis.

### 3.6. Physical Adhesion PDMS Patch

**Figure 6:**
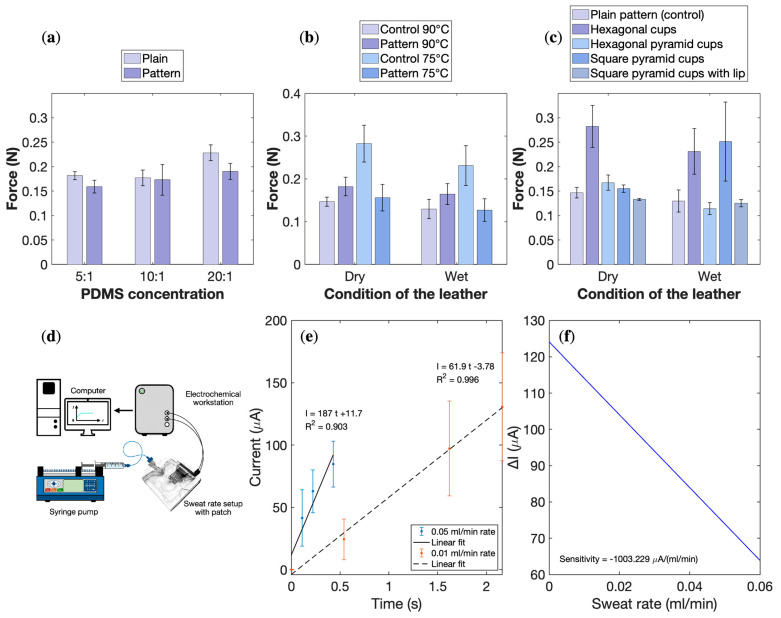
Adhesion performance of the PDMS patch and response of the sweat-rate sensor. (**a**) Detachment force for plain and hexagonal patterns at PDMS ratios of 5:1, 10:1, and 20:1 on a smooth copper surface. (**b**) Effect of curing temperature (75 °C vs. 90 °C) and pattern on adhesion under dry and wet conditions on imitation leather. (**c**) Comparison of alternative suction-cup geometries at 20:1 and 90 °C. (**d**) Experimental setup of the tLSG-based sweat-rate sensor with an overlying PDMS microchannel. (**e**) Chronoamperometric response to stepwise changes in artificial sweat flow rates of 0.01 and 0.05 mL/min. (**f**) Calibration plot of current change versus flow rate, yielding a sensitivity of approximately −1.0 mA·(mL/min)^−1^ within the tested range.

The attachment strength of the PDMS patches was evaluated using an Instron^®^ 5966 tensile testing machine equipped with a 10 N load cell and a small pneumatic clamp. Patches with different pattern designs (plain, hexagonal suction cups, and square cups) and PDMS mixing ratios (5:1, 10:1, 20:1) were tested on imitation leather substrates under both dry and wet conditions. Each patch was placed on a clean, level surface, clamped at the top edge, and gently pressed to ensure uniform contact before testing. The vertical extension rate was set to 10 mm/min, and the detachment force was defined as the first drop in the load–extension curve. For each pattern–concentration combination, three replicates were measured on the copper plate and five on the leather substrate, and the full experiment was repeated five times for each surface condition (Figure 6a–c).

In the first set of experiments, patches with a plain surface were compared to patches with a hexagonal suction-cup pattern at PDMS ratios of 5:1, 10:1, and 20:1 on the smooth copper surface. The average adhesion forces ranged from 0.1816 to 0.2282 N for plain patches and from 0.1591 to 0.1904 N for patterned patches, showing only a slight increase with increasing base: curing-agent ratio (Figure 6a). A two-way ANOVA on PDMS ratio (F = 2.70, *p* = 0.108), pattern (F = 2.07, *p* = 0.176), and their interaction (F = 0.43, *p* = 0.662) indicated no statistically significant effects, suggesting that neither concentration nor pattern had a dominant influence on adhesion in this configuration. Consequently, a 20:1 ratio was selected for subsequent tests as it provided convenient handling during fabrication and higher elasticity for further pattern optimization.

In the second set, plain and hexagonal-patterned patches with a 20:1 ratio were cured at 75 °C or 90 °C and tested on imitation leather under dry and wet conditions to emulate skin before and after sweating (Figure 6b). The average adhesion forces ranged from 0.1468 to 0.2825 N in the dry state and from 0.1272 to 0.2310 N in the wet state, with the highest values observed for the hexagonal pattern cured at 90 °C. At this temperature, patterned patches outperformed plain patches by approximately 92.5% under dry and 78% under wet conditions. A three-way ANOVA revealed marginally significant effects of pattern (F = 4.07, *p* = 0.052) and curing temperature (F = 3.48, *p* = 0.071), as well as a highly significant interaction between pattern and temperature (F = 12.28, *p* = 0.001), indicating that increasing the curing temperature from 75 °C to 90 °C substantially enhances the benefit of the hexagonal suction-cup geometry.

The final set of experiments compared multiple pattern designs at a fixed PDMS ratio of 20:1 and curing temperature of 90 °C, including a modified hexagonal cup with a pyramidal profile and square pyramid cups with and without an external lip (Figure 6c and Figure S9). Tests on dry and wet imitation leather showed that most alternative patterns performed worse than the straight hexagonal cups, with the exception of the square pyramid cups under wet conditions, which achieved an average adhesion force of 0.2512 N, comparable to 0.2310 N for the hexagonal design. A two-way ANOVA revealed a significant effect of pattern (F = 5.19, *p* = 0.0018), with follow-up one-way ANOVA indicating significance in dry conditions (F = 8.09, *p* = 0.00048) but not in wet conditions (F = 2.27, *p* = 0.098). Despite these trends, a Tukey HSD test did not find statistically significant pairwise differences between all groups, likely due to the relatively high variance, especially for the square pyramid design.

Overall, the adhesion tests identified the hexagonal suction-cup pattern fabricated with a 20:1 PDMS ratio and cured at 90 °C as a robust compromise between fabrication practicality and attachment strength. Under these conditions, the patches generated adhesion forces equivalent to approximately 28.8 g of supported weight in dry and 23.1 g in wet conditions, which is expected to be sufficient to carry the on-board electronics of the sweat patch during regular movement. Nonetheless, variability in patch thickness and curing homogeneity was observed and may have influenced the results more strongly than pattern geometry alone. Future work will therefore focus on improving process control and performing tests with more replicates and varied peeling angles to better approximate real-life wear scenarios.

### 3.7. Sweat Rate Sensor

A sweat-rate sensor was developed by integrating interdigital electrodes (IDEs) made from laser-scribed graphene with a PDMS-based microfluidic channel, operated in a chronoamperometric configuration (Figure 6d). The sensing principle is based on monitoring the change in electrical current between the IDE fingers as artificial sweat enters and progressively fills the microchannel above them. Because artificial sweat is an ionic medium, it decreases the resistance between adjacent electrode fingers as it bridges the gaps, resulting in a change in the measured current. As more fluid enters the channel, the resistance continues to evolve, enabling real-time tracking of the filling dynamics.

The sweat rate Q is related to the time required to fill a microchannel of known volume according to

(1) $$Q=\frac{V}{t}[µL/s]$$

where V is the fixed channel volume (0.36 µL) and t is the filling time. Stepwise chronoamperometric experiments were performed at controlled flow rates of 0.01 and 0.05 mL/min using artificial sweat, and the resulting current transients were recorded (Figure 6e). The correlation between the estimated flow rates and peak current responses showed good linearity, with coefficients of determination $R^{2}=0.99$ at 0.01 mL/min and $R^{2}=0.90$ at 0.05 mL/min.

From the calibration plot of current change versus flow rate (Figure 6f), the sensitivity of the sensor was determined to be approximately −1.0 mA·(mL/min)^−1^, indicating that the measured current decreases in magnitude as the imposed sweating rate increases. This counterintuitive trend may arise from rapid channel filling leading to earlier saturation of the conductive path, changes in effective ionic concentration and transport at higher flow rates, or geometrical effects related to the finite channel volume, and will require further investigation. Nevertheless, the linear relationship between flow rate and current response within the tested range confirms that the tLSG IDEs combined with the PDMS microchannel can transduce changes in sweat rate in a passive, non-enzymatic, and reagent-free manner, relying solely on the intrinsic conductivity of sweat [45,46]. These characteristics make the approach attractive for continuous sweat-rate monitoring in wearable applications.

### 3.8. Temperature Influence and Stability

The interference of common sweat constituents, such as sodium ions and urea, on the pH sensor performance was systematically investigated (Figure S10). To simulate more extreme chemical environments and ensure the robustness of the device, the interference tests were conducted in a 1 M PBS background (pH 7.2). The sensor exhibited a low cross-sensitivity of 7.12 mV/decade toward NaCl within the physiological range (1–100 mM). Furthermore, the response to urea (1–12 mM) was negligible, with a sensitivity of only 0.21 mV/decade and a low correlation coefficient R^2^ = 0.14 (Figure S11), indicating that the potential fluctuations were non-systematic and remained within the baseline noise. These results demonstrate the high selectivity of the developed pH sensor, ensuring minimal interference from the sweat matrix during real-time monitoring.

The thermal stability of the pH sensor was evaluated within the physiological range of 34–42 °C. Interestingly, the OCP response exhibited a non-monotonic trend, characterized by a potential increase up to 38 °C followed by a return to baseline levels at 42 °C (Figure S12). This behavior is attributed to the competitive temperature-dependent processes occurring at the ZnO/electrolyte interface and the LSG pseudo-reference electrode [44,47]. Such phenomena are well-documented for all-carbon electrochemical systems, where the absence of a conventional liquid junction leads to a complex but reproducible thermal drift that can be effectively compensated through second-order polynomial modeling [48].

To address the influence of skin temperature fluctuations on ion-selective sensor performance, the thermal stability of the ammonium ISE was evaluated as a model system across the physiological range (34–42 °C). As shown in Figure S13, the sensor exhibited a temperature-dependent response with sensitivities of 39.17, 33.25, and 29.17 mV/decade at 34, 38, and 42 °C, respectively. While the deviation from the theoretical Nernstian slope at elevated temperatures is often observed in flexible membrane-based sensors due to altered ion-partitioning and diffusion kinetics [19,49,50], the response remained highly linear (R^2^ > 98). This predictability allows for accurate real-time pH and ion concentration recovery by integrating data from the on-patch temperature sensor for software-based compensation.

## 4. Discussion

The results demonstrate that transferring laser-scribed graphene onto PDMS yields electrodes with stable electrochemical behavior and adequate conductivity for sweat-sensing applications. The combination of porous LSG and compliant PDMS provides a favorable balance between charge-transfer performance and mechanical flexibility, enabling conformal skin contact without compromising electrode stability. These characteristics position the tLSG-on-PDMS platform as a viable basis for integrated wearable biosensors.

By integrating temperature, pH, ammonium, potassium, sodium, and sweat rate sensing into a single patch, the device enables multiplexed monitoring of physiologically relevant parameters in sweat. The obtained sensitivities for NH_4_^+^, K^+^, and pH, together with the linear temperature response and flow-dependent current in the sweat-rate channel, confirm that the platform can capture dynamic changes in local biochemical and physical conditions during exercise. Although the sodium-selective electrode currently exhibits strongly sub-Nernstian behavior, it illustrates the feasibility of implementing additional ion channels on the same tLSG substrate and identifies clear directions for membrane optimization.

The development of the present platform was motivated by, and tested in the context of, an adaptive cycling marathon across Saudi Arabia, during which a prototype version of the patch was used to stream sweat-related signals online. Although detailed field data from that campaign are beyond the scope of this work, the experience informed the mechanical and integration requirements for the final tLSG-on-PDMS design. Further information about the campaign is available from the public Athar project documentation (KAUST, 2023) [34].

**Figure 7:**
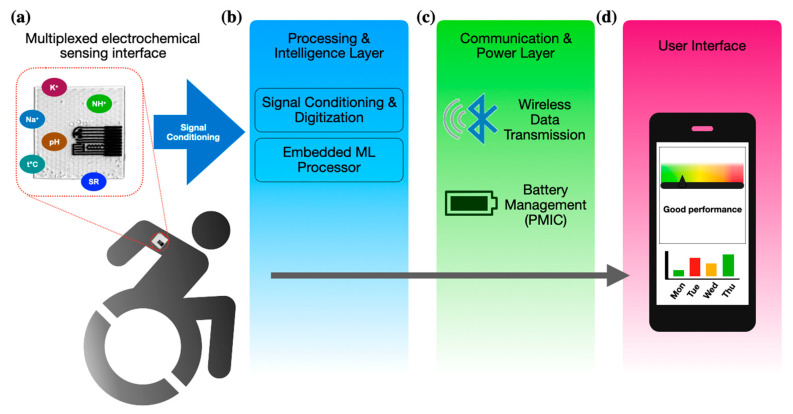
Proposed system architecture for the integrated wearable platform, illustrating the signal pathway from the LSG-based sensors to the data processing and wireless transmission modules: (**a**) the multiplexed sensor patch that captures real-time biochemical signals; (**b**) an embedded processing unit that performs signal conditioning and runs a TinyML algorithm to infer the athlete’s physiological exertion level; and (**c**) a wireless communication interface that transmits the processed telemetry to (**d**) a dashboard for real-time visualization. This modular design ensures that the sensor platform is scalable for standalone wearable applications.

To demonstrate the feasibility of the developed platform for autonomous wearable applications, a conceptual system-level architecture was designed (Figure 7). This architecture outlines the signal path from the multiplexed electrochemical sensing interface to the integrated data processing and wireless communication modules, confirming the platform’s suitability for continuous real-time physiological monitoring.

Despite these promising results, several limitations remain. The sodium-selective membrane requires reformulation to approach Nernstian sensitivity and improve selectivity and adhesion on the tLSG surface, and the sweat-rate sensor behavior at higher flow rates needs further investigation to fully clarify the origin of its negative current slope. In addition, the present study relies primarily on artificial sweat for calibration and does not yet include systematic benchmarking against gold-standard clinical measurements in real human sweat. Future work will focus on ion-selective membrane optimization, long-term drift and fouling studies in vivo, wireless integration for untethered data collection, and more detailed physico-chemical characterization of the LSG–PDMS interface.

Regarding thermal performance, while our results confirm that the device effectively measures biochemical parameters across the physiological temperature range, the observed temperature-dependent sensitivity of the ammonium ISE highlights a fundamental limitation: the potential for measurement error if ambient temperature is not accounted for. Although the sensor response is highly linear and predictable, which facilitates post-processing compensation, the reliance on such software-based corrections requires precise synchronization between the ion-selective channels and the on-patch temperature sensor. Future optimization should prioritize the development of more thermally robust membrane compositions or the implementation of autonomous, on-chip temperature-compensating circuitry to mitigate these sensitivities directly at the hardware level.

It should be noted that while the sweat-rate sensor utilizes the conductivity of sweat to transduce channel filling, the estimation of flow rate (Q = V/t) is primarily derived from the temporal dynamics of the filling process (i.e., the time interval between successive electrode bridges). This temporal approach is inherently less sensitive to fluctuations in absolute ionic concentration compared to magnitude-based conductivity measurements. Nevertheless, to ensure robust operation under varying physiological electrolyte concentrations, the concurrent data from our integrated Na^+^ and K^+^ sensors can be used for real-time normalization of the ionic background, allowing for effective cross-verification and decoupling of electrolyte-dependent conductivity variations from the volumetric sweat rate signal.

## 5. Conclusions

In this work, a proof-of-concept wearable sweat patch based on laser-scribed graphene transferred onto PDMS was developed and characterized as an integrated platform for multiplexed sensing. The tLSG-on-PDMS electrodes exhibited stable electrochemical performance and sufficient conductivity while maintaining the mechanical flexibility and biocompatibility required for prolonged skin contact. Together, these properties establish a practical foundation for soft, skin-conformal sweat biosensors.

The device combines temperature, pH, ion-selective, and sweat-rate sensing in a single patch, enabling simultaneous monitoring of multiple physiologically relevant parameters. The ammonium- and potassium-selective electrodes, pH sensor, and temperature channel demonstrated reproducible, linear responses within relevant concentration and temperature ranges, while the sweat-rate sensor provided a flow-dependent current signal suitable for tracking changes in perspiration dynamics. Although the sodium-selective electrode currently shows strongly sub-Nernstian behavior, it highlights the feasibility of adding further ion channels to the same tLSG platform and identifies clear targets for membrane optimization.

A custom PDMS adhesive patch with suction-cup-inspired microstructures was engineered to enhance attachment on skin-like substrates under dry and wet conditions. Adhesion tests revealed that the optimized hexagonal pattern, combined with an appropriate PDMS mixing ratio and curing temperature, can generate sufficient detachment forces to support the weight of the integrated electronics during movement. This mechanical robustness, together with the softness of PDMS, is crucial for stable, long-term wearable operation without compromising user comfort.

Finally, the system was deployed during an adaptive cycling marathon across Saudi Arabia, providing an initial demonstration of robustness and feasibility in a demanding real-world scenario. The platform withstood prolonged use, repeated mechanical deformation, and varying sweating conditions, underscoring its potential for sports performance monitoring and rehabilitation-focused applications. Future efforts will concentrate on improving ion-selective membrane formulations, refining sweat-rate transduction at higher flow regimes, validating sensor outputs in real sweat against clinical standards, and integrating wireless electronics to realize a fully autonomous, multiplexed wearable sweat monitoring system.

## Figures and Tables

**Table 1:** Cathodic peak current (Ipc) and Anodic peak current (Ipa) of the electrodes.

Electrode	Ipa (μA)	σ %	Ipc (μA)	σ %
Left	9	9.70	−9	8.08
Middle	13	6.99	−15	4.96
Right	11	3.68	−13	9.60

## Data Availability

Data will be made available upon request.

## Supplementary Materials

The following supporting information can be downloaded at https://www.mdpi.com/article/10.3390/bios16050277/s1. Figure S1: The process of LSG on PDMS transfer; Figure S2: Electrical conductivity of tLSG; Figure S3: Stability test of tLSG: 3 electrode system; Figure S4: Reproducibility test of tLSG; Figure S5: Energy Dispersive X-ray Spectrometry (EDS) of tLSG and LSG sputter-coated with a 3 nM layer of Iridium; Figure S6: Sensitivity test of Ammonium selective electrode; Figure S7: Sensitivity test of Potassium selective electrode; Figure S8: Sensitivity test of Sodium selective electrode; Figure S9: Different suction-cup geometries; Figure S10: Impact of ionic strength on pH sensor stability; Figure S11: Anti-interference performance of the LSG-based pH sensor; Figure S12: Thermal stability and compensation model for the pH sensor using an LSG pseudo-reference electrode; Figure S13: Temperature-dependent performance of the ammonium-selective sensor; Figure S14: Cross-section Scanning electron microscopy (SEM) images.

## Abbreviations

The following abbreviations are used in this manuscript:

LSG	Laser-scribed graphene
tLSG	Transferred laser-scribed graphene
PDMS	Polydimethylsiloxane
PVC	Polyvinyl chloride
THF	Tetrahydrofuran
PI	Polyimide
WE	Working electrode
OCP	Open circuit potential
IDE	Interdigital electrodes
CV	Cyclic voltometry
Ipa	Anodic peak currents
Ipc	Cathodic peak current
TCR	Temperature coefficient of resistance
