# Supplementary material for: Laser-Scribed Graphene on PDMS for Flexible Wearable Sweat Biosensors with Multiplexed Sensing Capability
Source: Biosensors (Basel). 2026 May 11;16(5):277. doi: 10.3390/bios16050277 (PMC13204617; doi:10.3390/bios16050277)
Supplement: Supplementary file 1 [file biosensors-16-00277-s001.zip › biosensors-4262393-supplementary.pdf]

biosensors

MDPI

Supplementary Materials

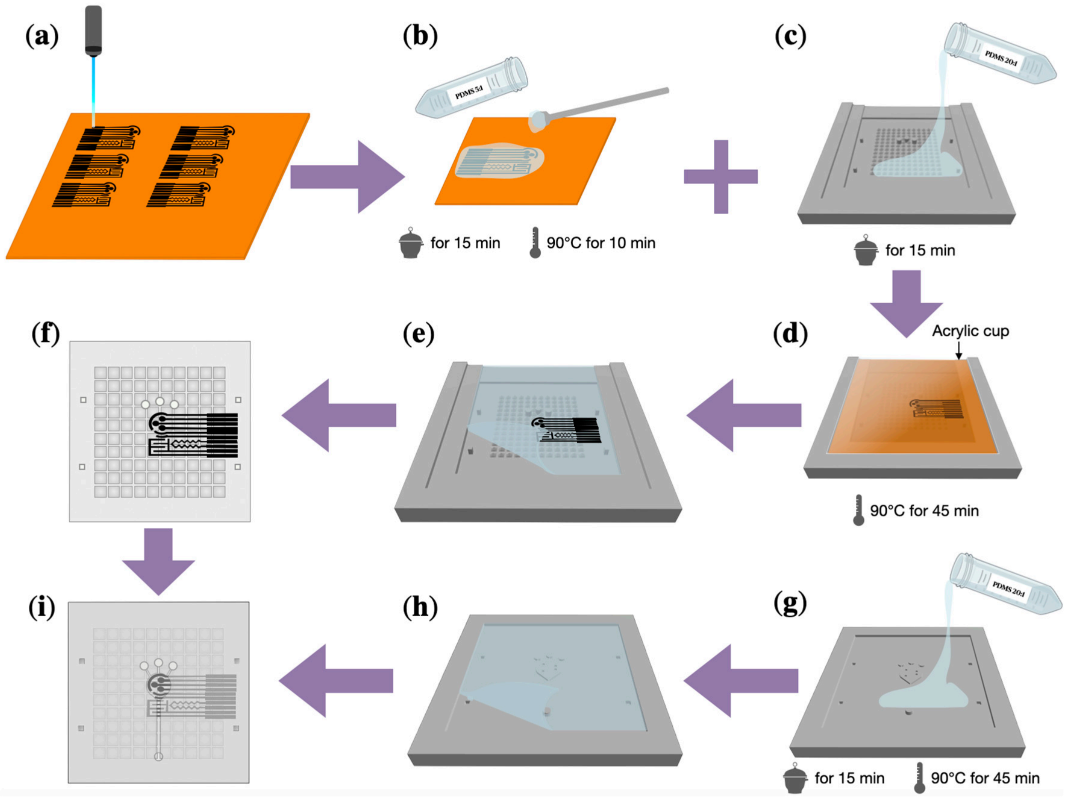

Figure S1. The process of LSG on PDMS transfer. (**a**) CO2 Laser machine heats polyamide sheet; (**b**) applies PDMS on LSG; (**c**) pour PDMS on LSG in mold; (**d**) merge parts; (**e**) peeling off PDMS; (**f**) tLSG; (**g**) pour PDMS in Microfluidic (MF) mold; (**h**) peeling off PDMS MF; (**i**) arrange layers of tLSG and MF.

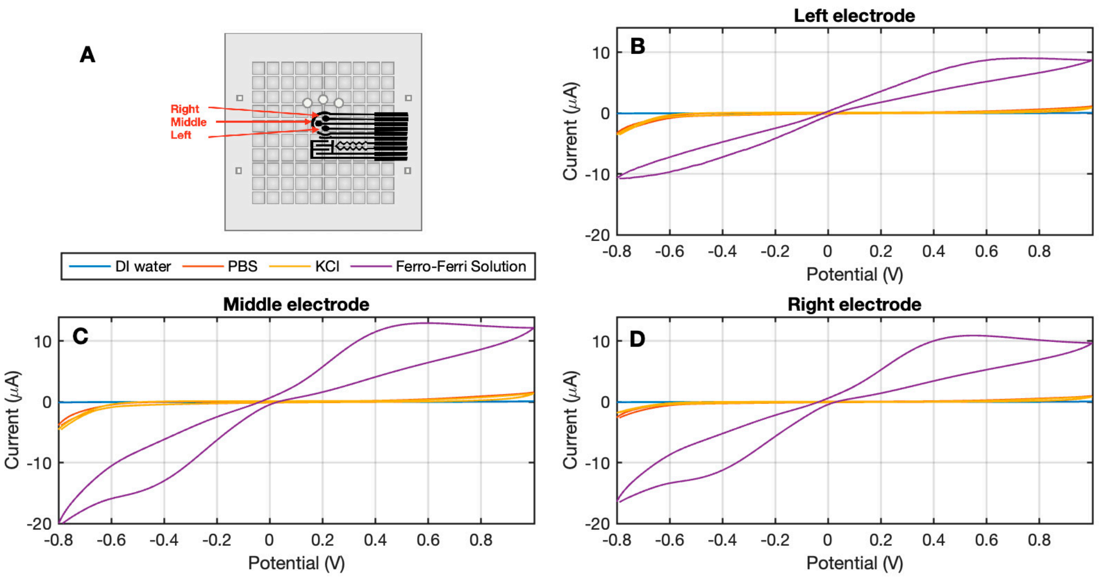

Figure S2. (**a**) Schematical label of patch electrodes; Electrical conductivity test for left (**b**), middle (**c**) and right electrodes (**d**): 3 electrode system, 10 mM Ferro-Ferri in 10 mM PBS; Electrical conductivity: ~ 0.06 S/m for each electrode.

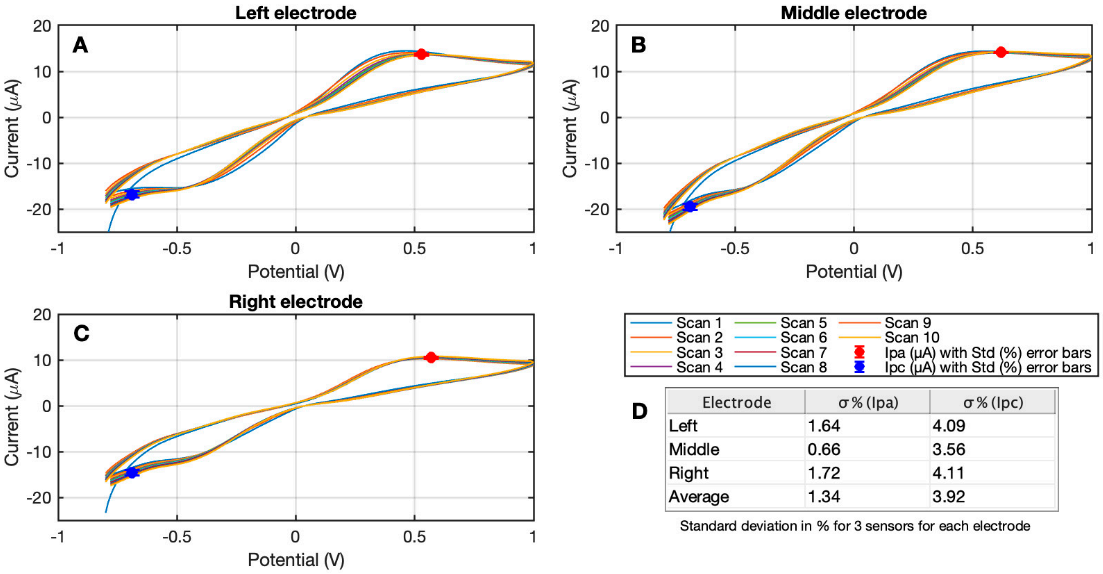

Electrode	σ % (Ipa)	σ % (Ipc)
Left	1.64	4.09
Middle	0.66	3.56
Right	1.72	4.11
Average	1.34	3.92

Figure S3. Stability test of tLSG: 3 electrode system, 10 mM Ferro-Ferricyanide solution in 10 mM PBS Electrical conductivity: ~ 0.06 S/m for left (**a**) middle (**b**) and right (**c**) electrodes with a STD in percents in table (**d**).

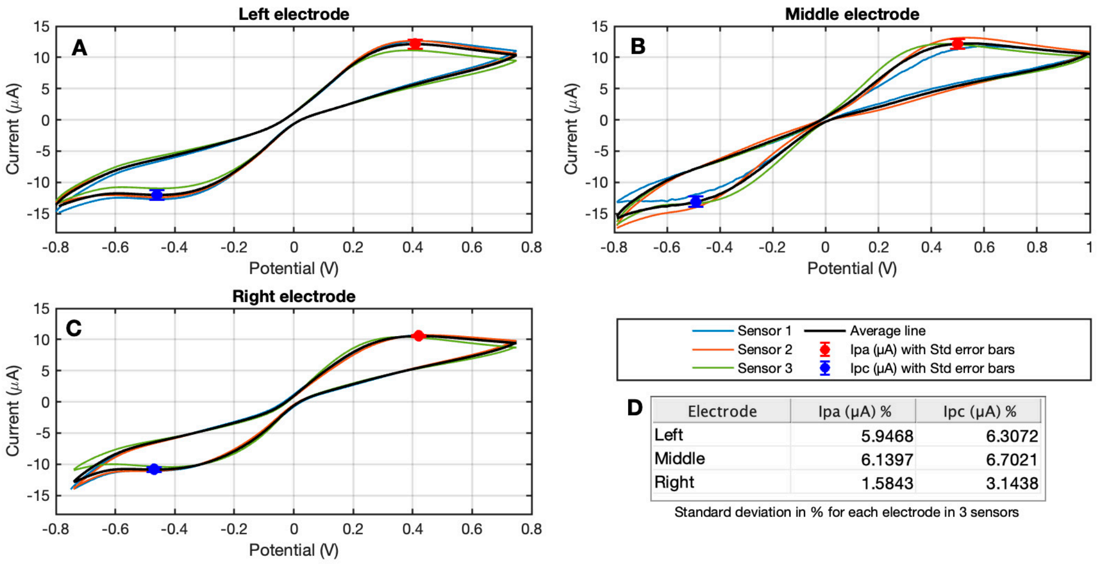

Electrode	Ipa (μA) %	Ipc (μA) %
Left	5.9468	6.3072
Middle	6.1397	6.7021
Right	1.5843	3.1438

Standard deviation in % for each electrode in 3 sensors

Figure S4. Reproducibility test of tLSG: (**a**) left electrode's STD - 6%; (**b**) middle electrode's STD - 6%; (**c**) right electrode's STD - 1%; (**d**) STD in 3 sensors on each electrode in percents in table.

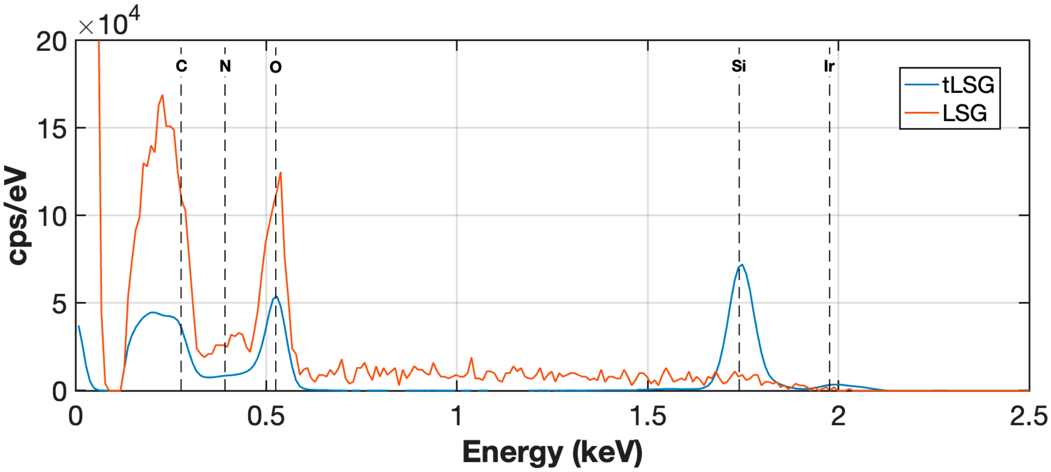

Figure S5. Energy Dispersive X-ray Spectrometry (EDS) of tLSG and LSG sputter-coated with a 3 nM layer of Iridium.

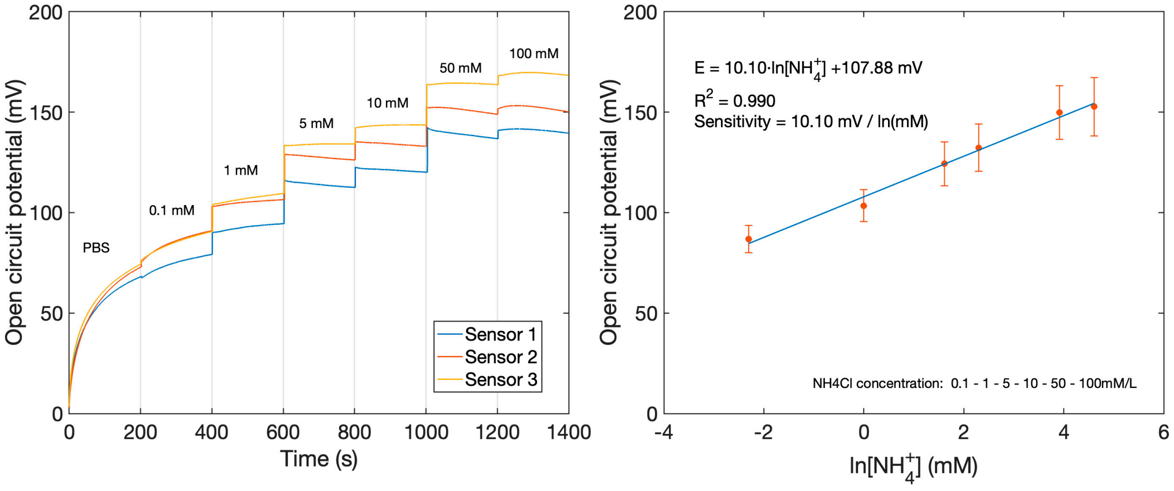

(**a**) (**b**)

Figure S6. Sensitivity test of Ammonium selective electrode: (**a**) Calibration curve Open circuit potential vs time with different concentration of NH4Cl (n = 3); (**b**) Sensitivity graph - 10.10mV/ln[NH_3+] with Std dev (n = 3).

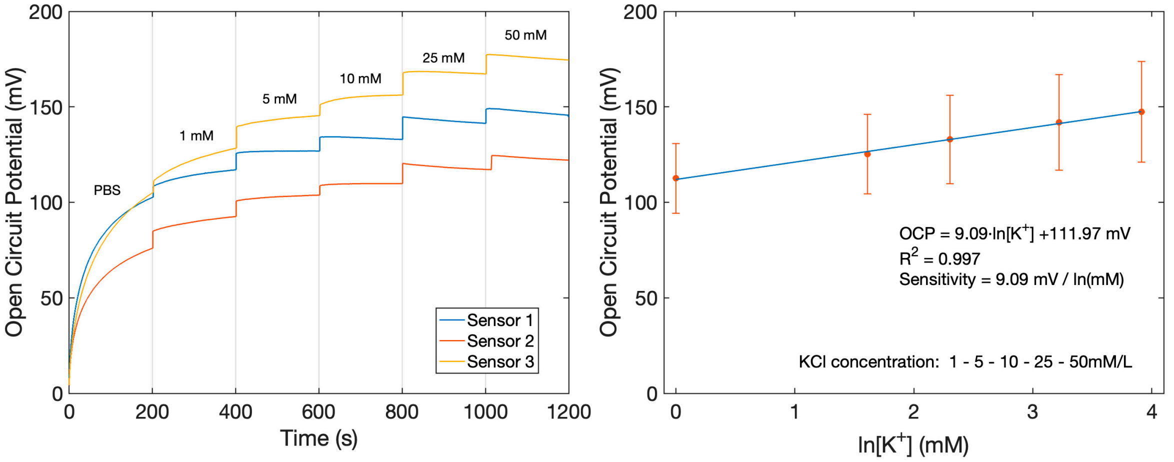

(**a**) (**b**)

Figure S7. Sensitivity test of Potassium selective electrode: (**a**) Calibration curve Open circuit potential vs time with different concentration of KCl (n = 3); (**b**) Sensitivity graph - 9.3mV/ln[K+] with Std dev (n = 3).

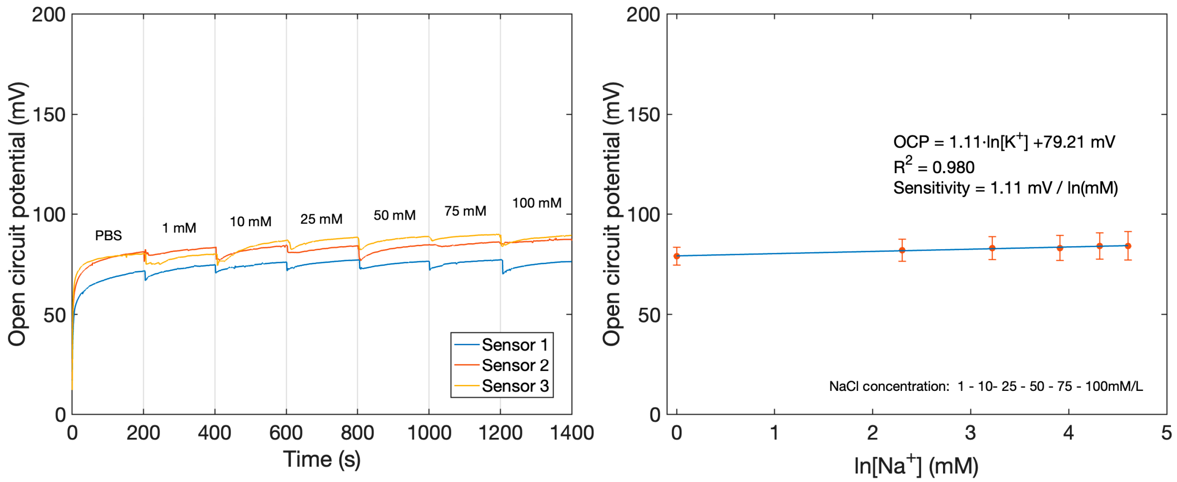

(**a**) (**b**)

Figure S8. Sensitivity test of Sodium selective electrode: (**a**) Calibration curve Open circuit potential vs time with different concentration of NaCl (n = 3); (**b**) Sensitivity graph - 1.11mV/ln[Na+] with Std dev (n = 3).

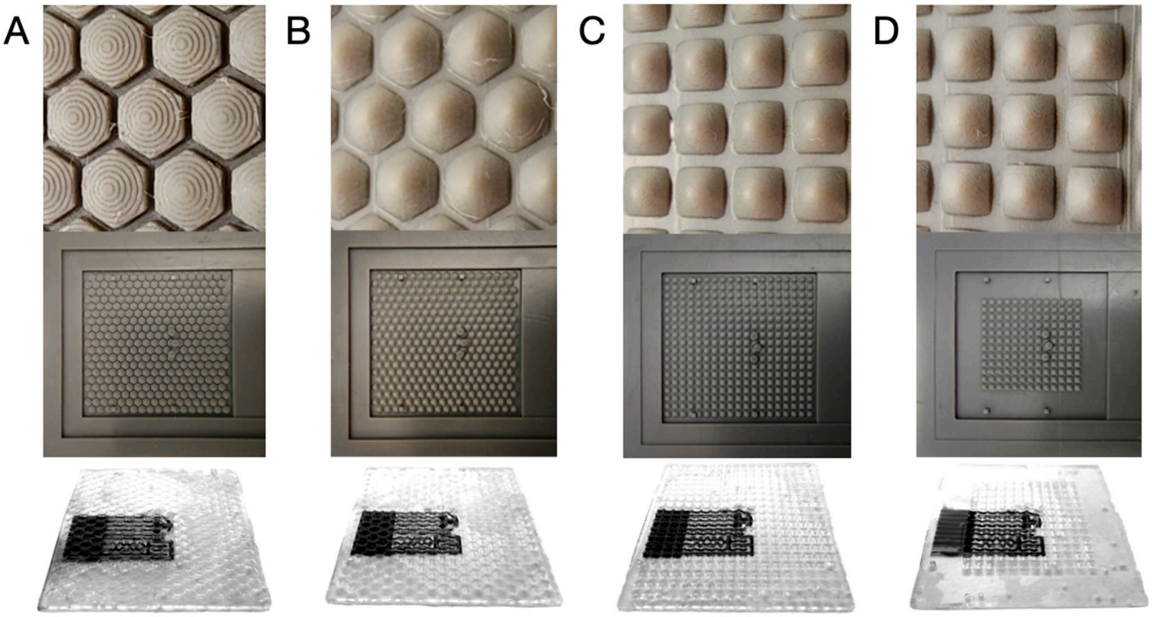

Figure S9. Different suction-cup geometries: (**a**) Hexagonal cups; (**b**) Hexagonal pyramid cups; (**c**) Square pyramid cups; (**d**) Square pyramids cup with lip.

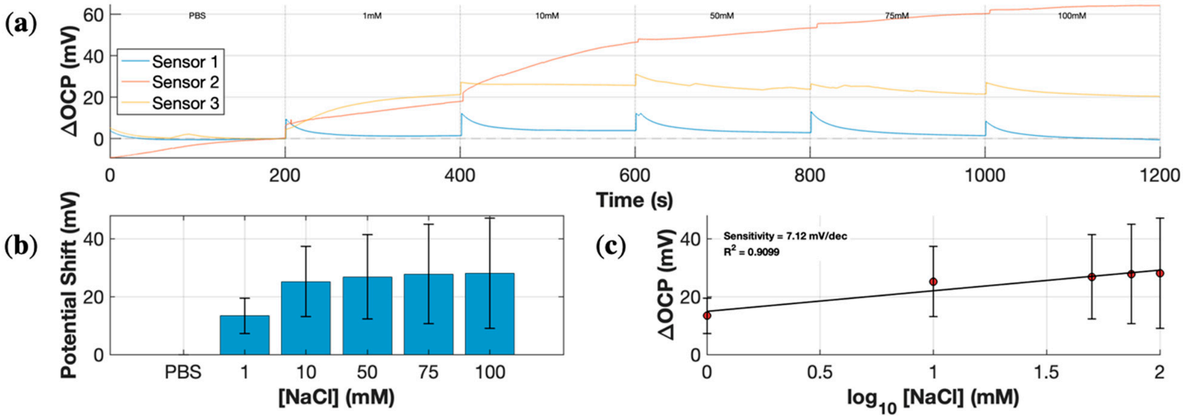

Figure S10. Impact of ionic strength on pH sensor stability. (**a**) Normalized dynamic OCP response to stepwise increments of NaCl concentration (1–100 mM). (**b**) Mean potential drift as a function of [NaCl] concentration. (**c**) Linear regression of the potential shift vs. log-concentration, showing a sensitivity of 7.12 mV/decade (R^2=0.91).

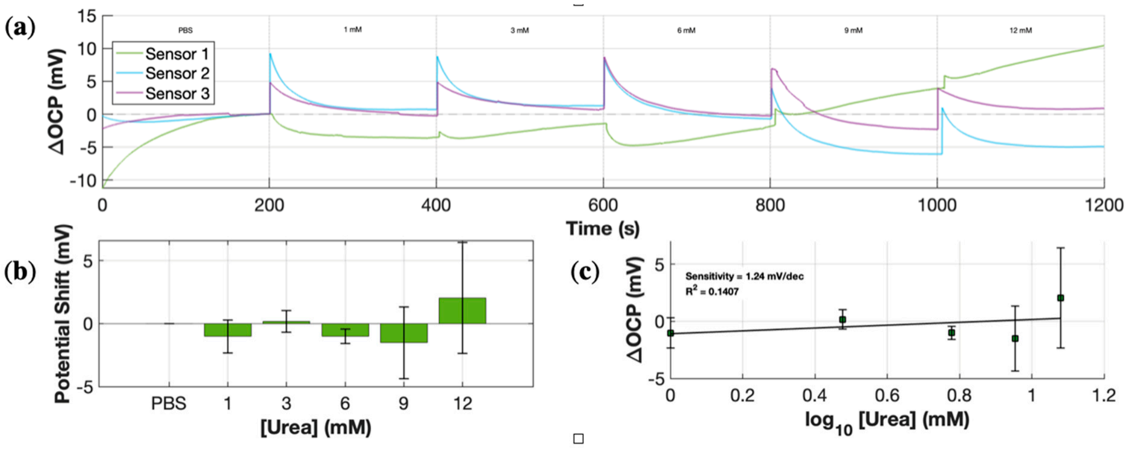

Figure S11. Anti-interference performance of the LSG-based pH sensor. (**a**) Calibration curve for Urea interference. (**b**) Dynamic OCP response to increasing urea concentrations (1–12 mM) in 1M PBS. (**c**) Calibration curve for urea interference showing a negligible sensitivity of 1.24 mV/decade.

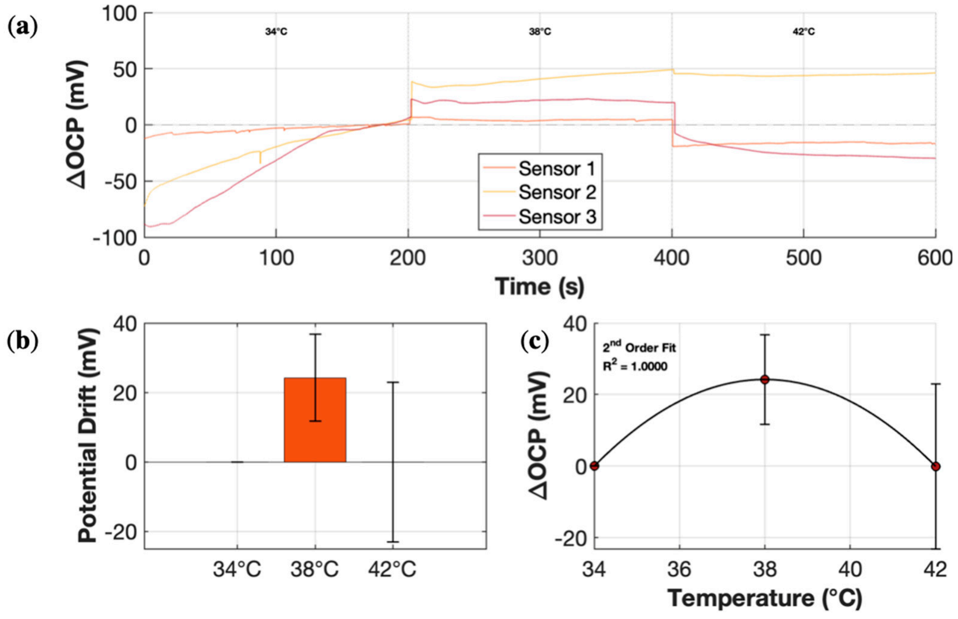

Figure S12. Thermal stability and compensation model for the pH sensor using an LSG pseudo-reference electrode. (**a**) Dynamic OCP response recorded at a fixed pH of 6.2 under increasing temperatures (34, 38, and 42 °C). (**b**) Mean potential drift relative to the 34 °C baseline. (**c**) Non-linear thermal compensation model based on a second-order polynomial fit (R^2 =1). The quadratic trend accounts for the overlapping thermal coefficients of the ZnO sensing interface and the graphene double-layer capacitance, enabling accurate real-time software-based compensation.

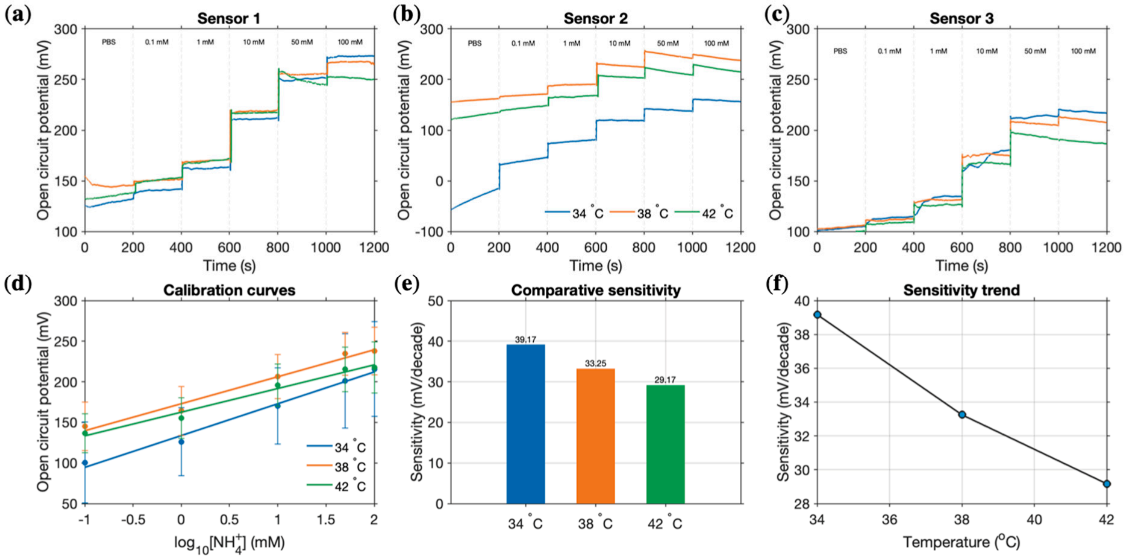

Figure S13. **Temperature-dependent performance of the ammonium-selective sensor.** (**a-c**) Real-time open circuit potential response of the sensor to increasing NH_4^+ concentrations (n=3) at 34 °C, 38 °C, and 42 °C. (**d**) Corresponding calibration curves showing excellent linearity across the physiological temperature range. (**e**) Summary of comparative sensitivity (mV/decade) at each temperature, illustrating the Nernstian behavior where slope decreases with increasing temperature. (**f**) Sensitivity trend highlighting the predictable thermal dependency, facilitating potential on-chip temperature compensation for reliable wearable sweat monitoring.

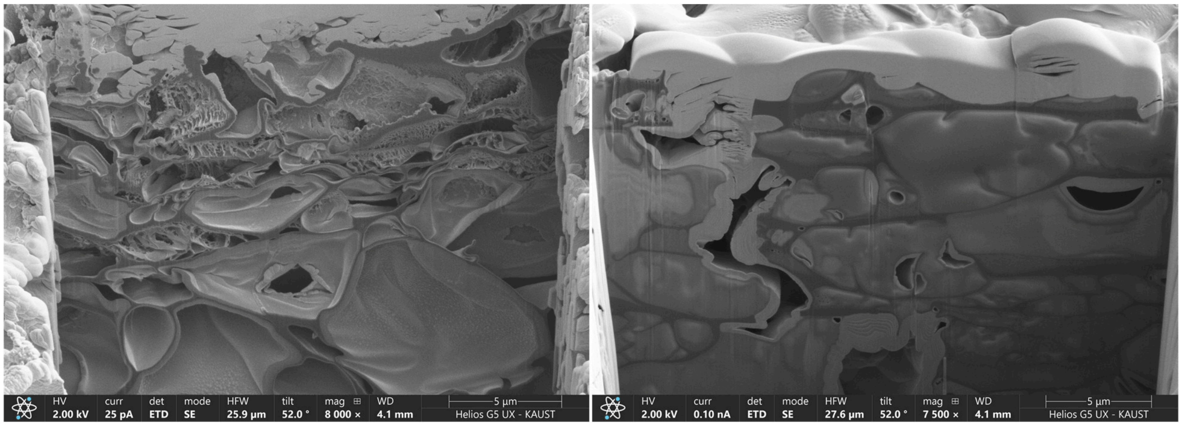

(**a**) (**b**)

Figure S14. Cross-section Scanning electron microscopy (SEM) images of: (a) the laser-scribed graphene electrode fabricated on polyimide film; (b) sponge-like internal morphology of transferred laser-scribed graphene on PDMS.
